# Telomere maintenance and the DNA damage response: a paradoxical alliance

**DOI:** 10.3389/fcell.2024.1472906

**Published:** 2024-10-17

**Authors:** Ashley Harman, Tracy M. Bryan

**Affiliations:** Cell Biology Unit, Children’s Medical Research Institute, Faculty of Medicine and Health, University of Sydney, Westmead, NSW, Australia

**Keywords:** telomere maintenance, telomerase, shelterin, DNA damage response, replication stress, telomere replication, nuclear actin

## Abstract

Telomeres are the protective caps at the ends of linear chromosomes of eukaryotic organisms. Telomere binding proteins, including the six components of the complex known as shelterin, mediate the protective function of telomeres. They do this by suppressing many arms of the canonical DNA damage response, thereby preventing inappropriate fusion, resection and recombination of telomeres. One way this is achieved is by facilitation of DNA replication through telomeres, thus protecting against a “replication stress” response and activation of the master kinase ATR. On the other hand, DNA damage responses, including replication stress and ATR, serve a positive role at telomeres, acting as a trigger for recruitment of the telomere-elongating enzyme telomerase to counteract telomere loss. We postulate that repression of telomeric replication stress is a shared mechanism of control of telomerase recruitment and telomere length, common to several core telomere binding proteins including TRF1, POT1 and CTC1. The mechanisms by which replication stress and ATR cause recruitment of telomerase are not fully elucidated, but involve formation of nuclear actin filaments that serve as anchors for stressed telomeres. Perturbed control of telomeric replication stress by mutations in core telomere binding proteins can therefore cause the deregulation of telomere length control characteristic of diseases such as cancer and telomere biology disorders.

## 1 Introduction

**FIGURE 1:**
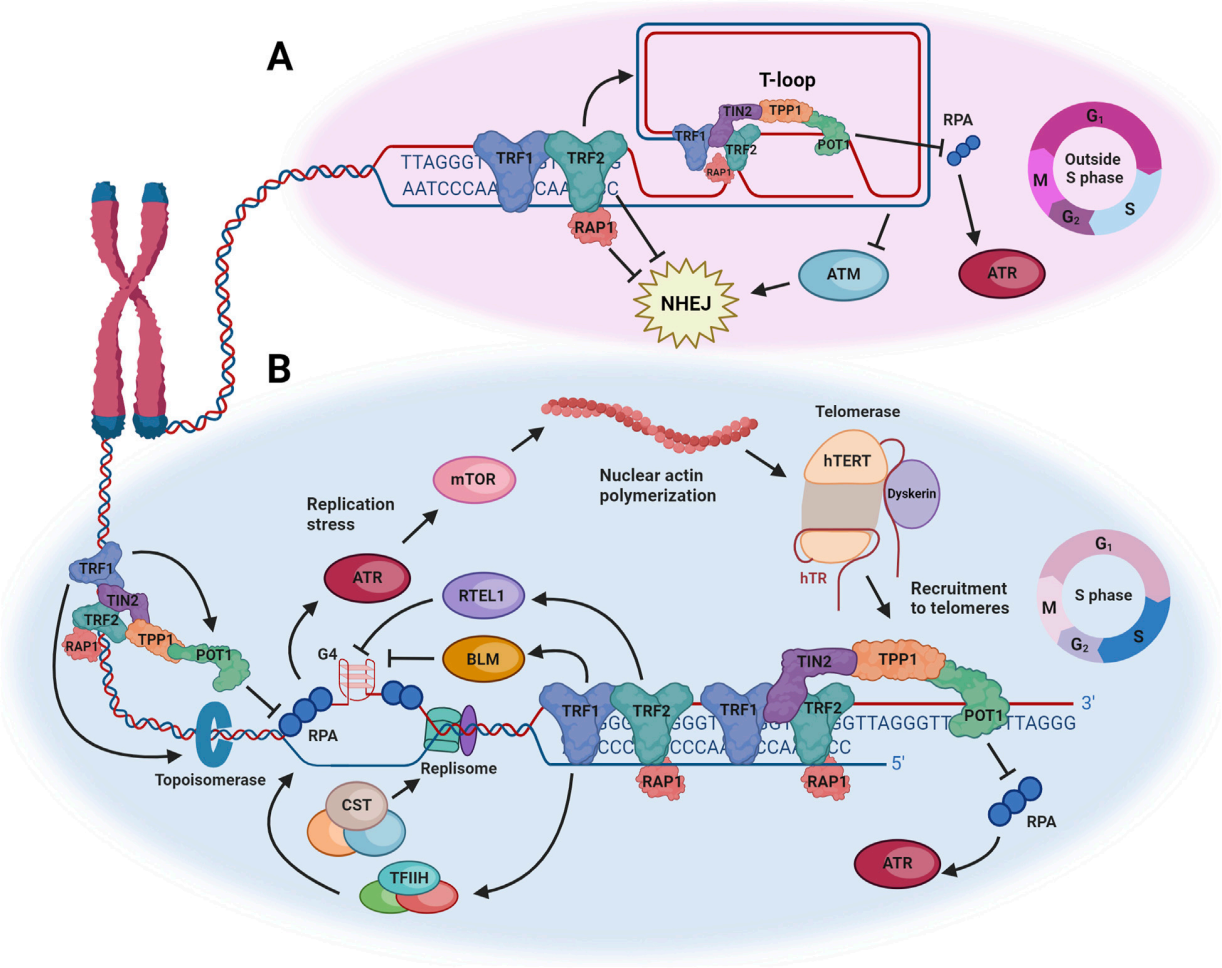
Summary of mechanisms by which the DNA damage response is repressed at telomeres or harnessed to facilitate telomerase-mediated telomere maintenance. **(A)** Shelterin protects telomeres from inappropriately activating a DNA damage response in phases of the cell cycle when the telomere can fold into a t-loop. TRF2 promotes t-loop formation, which prevents activation of ATM, and TRF2 also directly suppresses NHEJ. POT1 protects against activation of ATR. **(B)** During S phase, the t-loop is unwound to allow replication of the telomere by the canonical replication machinery, which is impeded by the repetitive sequence of telomeres, their occupation by shelterin, and propensity to form G-quadruplexes (G4). Multiple components of shelterin counteract the resulting replication stress, but any remaining stress activates ATR, which facilitates the polymerization of nuclear actin and ultimately the recruitment of telomerase to telomeres. See text for more details. Figure created with Biorender.com.

Telomeres are nucleoprotein complexes located at the ends of linear chromosomes which serve to maintain genomic integrity and ensure cellular survival. Telomeres were first identified in fruit flies and corn (McClintock, 1941; Muller, 1938) and have since been characterized in a range of eukaryotes. Human telomeres are comprised of tandem TTAGGG repeats which extend for 3–18 kb (Moyzis et al., 1988), ending in a single-stranded G-rich overhang 12–400 nucleotides long (Makarov et al., 1997; McElligott and Wellinger, 1997; Zhao et al., 2008). This overhang, which if exposed would resemble DNA damage, can strand invade the double-stranded region of the telomere to form a telomere-loop (t-loop; Figure 1A), which serves to protect the telomere from being misrecognized as a double-strand break (DSB) and activating a DNA damage response (DDR) (Doksani et al., 2013; Griffith et al., 1999; Van Ly et al., 2018).

Telomeric DNA is bound by a six protein complex named shelterin, which helps maintain telomere structure and function in many different ways (de Lange, 2005; de Lange, 2018; Figure 1). Shelterin binds to telomeres through TRF1 and TRF2 (telomere-repeat binding factor 1 and 2), which both recognize and bind to the double-stranded portion of the telomere (Bilaud et al., 1997; Broccoli et al., 1997; Chong et al., 1995). The telomere is also bound by POT1 (protection of telomere 1) which forms a heterodimer with TPP1 (TINT1/PTOP/PIP1) (Houghtaling et al., 2004; Liu et al., 2004; Ye et al., 2004b) and binds to the single-stranded overhang specifically through its oligonucleotide/oligosaccharide binding (OB) folds (Baumann and Cech, 2001; Loayza and De Lange, 2003). Rap1 is a conserved shelterin subunit and is recruited to telomeres by forming a complex with TRF2 (Li et al., 2000; Sfeir et al., 2010). The final shelterin component is TIN2 (TRF1-interacting factor 2) which bridges TRF1/2 and the TPP1-POT1 complex and helps to stabilize the latter in addition to the TRF2-Rap1 complex (Houghtaling et al., 2004; Kim et al., 1999; Liu et al., 2004; Ye and de Lange, 2004; Ye et al., 2004a). Shelterin is crucial for telomere protection, as loss of shelterin binding leads to deprotection of telomeres, unwinding of the t-loop and activation of at least seven different DDR pathways (de Lange, 2018; Sfeir and de Lange, 2012), culminating in genomic instability, cell cycle arrest and cell death. Conversely, the shelterin complex can also harness several DDR pathways to assist in maintenance of telomere length and avoidance of the cell cycle arrest and cell death that result from critically short telomeres. This article will discuss the complicated and paradoxical relationship between telomeres, the DDR, and telomere maintenance by the ribonucleoprotein enzyme telomerase, with a focus on events in human cells.

## 2 DNA damage and its suppression at the telomere

Given that the structure of a telomere resembles damaged DNA, it is imperative that the DDR is suppressed to prevent unnecessary repair of the telomere which could result in chromosome end-to-end fusions. Global DNA damage is detected largely by three phosphatidylinositol 3-kinase-related protein kinases (PIKKs): ATR (ataxia telangiectasia and Rad3-related), ATM (ataxia telangiectasia mutated) and DNA-PK (DNA-dependent protein kinase) (Blackford and Jackson, 2017). These kinases are responsible for the activation of various signaling pathways which trigger cell cycle arrest and the promotion of DNA repair mechanisms to resolve the DNA damage (Harrison and Haber, 2006). Should the extent of damage be irreparable then the kinases can instead signal the cell to undergo apoptosis or senescence (Nowsheen and Yang, 2012). ATR is activated in response to single-stranded DNA, which can occur at replication forks that are slowed or stalled in situations where DNA replication is impeded (a state known as “replication stress”) (Saldivar et al., 2017; Yang et al., 2004). Conversely, both ATM and DNA-PK can be activated in response to DSBs, although they each employ different pathways to repair the damage, through homology-directed repair or non-homologous end joining (NHEJ) respectively (Davis et al., 2014; Shiloh and Ziv, 2013).

The components of shelterin both directly and indirectly suppress an unwanted telomeric DDR. Indirect suppression relies on t-loop formation, as telomeres in this closed state are unlikely to trigger a DDR (Figure 1A). This conformation would prevent the telomere end from being detected by factors which sense exposed DNA ends and activate the DDR, such as MRN (Mre11, Rad50, Nbs1) or the Ku70/80 complex (Gottlieb and Jackson, 1993; Lee and Paull, 2005). The key regulator of t-loop formation is TRF2, as its depletion from cells results in reduced t-loop formation (Doksani et al., 2013; Van Ly et al., 2018). Furthermore, TRF2 can promote strand invasion *in vitro*, as it binds to DNA and promotes formation of structures which resemble t-loops (Amiard et al., 2007; Stansel et al., 2001). As telomeres shorten, or following moderate TRF2 depletion, t-loops unfold and telomere ends become accessible to the DNA damage sensing machinery. These are termed “intermediate-state” telomeres (Cesare and Karlseder, 2012; Cesare et al., 2009), which trigger a DDR predominately through ATM (Karlseder et al., 2004; Van Ly et al., 2018), leading to cell cycle arrest and senescence. However, critically short telomeres or extreme loss of TRF2 instead results in deprotected telomeres (an “uncapped-state”) which undergo ATM-dependent end-to-end chromosomal fusions, resulting in genome wide instability (Celli and de Lange, 2005; Denchi and de Lange, 2007). This is a consequence of TRF2 also directly repressing the downstream consequences of ATM activation at telomeres, via a short region named the iDDR (inhibitor of DDR) within the hinge domain of TRF2 (Okamoto et al., 2013). This motif inhibits telomeric localization of the E3 ubiquitin ligase RNF168, in turn preventing 53BP1 accumulation at telomeres and chromosomal fusion via NHEJ (Okamoto et al., 2013).

On the other hand, ATR activation resulting from an exposed single-stranded telomeric overhang is thought to be primarily suppressed by POT1, since POT1 depletion activates ATR and results in ATR-dependent DNA damage signaling at telomeres (Denchi and de Lange, 2007; Figures 1A, B). This is dependent on the ability of POT1 to bind to telomeres, as preventing its recruitment via TPP1 or TIN2 inhibition also results in ATR activation (Hockemeyer et al., 2007; Takai et al., 2011). POT1 binds to single-stranded telomeric repeats, including the 3′telomeric overhang, preventing binding of RPA (replication protein A) (Gong and de Lange, 2010), which coats single-stranded DNA and is a key factor in the activation of ATR (Zou and Elledge, 2003).

## 3 Shelterin suppression of the DDR resulting from telomere replication

While the structural similarity of exposed telomeres and damaged DNA suggests that telomere-associated proteins would strive to keep DDR proteins away, paradoxically this does not always appear to occur. In fact, many DDR proteins are recruited to telomeres, not just in cancer cells, but also in normal somatic cells. For example, ATR localizes to telomeres during S phase in normal human fibroblasts (Verdun and Karlseder, 2006), and is necessary to protect telomere fidelity in both human and mouse cells (McNees et al., 2010; Pennarun et al., 2010). One possible explanation for this phenomenon is that telomeres are difficult regions for the DNA replication machinery to efficiently and correctly traverse, as repetitive DNA sequences (termed “fragile sites”) are highly prone to stalling replication machinery at the replication fork. Replication fork pausing or stalling has been observed in telomeres from yeast to humans (Fouche et al., 2006; Ivessa et al., 2002; Makovets et al., 2004; Sfeir et al., 2009), and may be further compounded at telomeres by shelterin binding also impeding DNA replication (Ohki and Ishikawa, 2004). Additionally, given their G-rich composition, telomeres readily form G-quadruplexes (G4s), DNA secondary structures which can also impede replisome progression (Bochman et al., 2012; Bryan, 2020). The inhibition of replication fork progression requires activation of processes to stabilize, repair and ultimately restart the replication fork. This response is regulated by ATR, which is activated by RPA-coated single-stranded DNA at the stalled replication fork (Shechter et al., 2004). Excessive replication stress at telomeres which cannot be resolved results in a “fragile telomere” phenotype: telomeres with breaks which appear as elongated or multiple signals at the ends of metaphase chromosomes (Sfeir et al., 2009). Given this, it is imperative that replication of the telomere is regulated to ensure its completion to prevent significant loss of telomeric DNA.

To prevent the generation of replication stress, shelterin helps to promote efficient telomere replication [reviewed in Bonnell et al. (2021); Higa et al. (2017a); Figure 1B]. The promotion of telomere replication by shelterin proteins is conserved across evolution, since Taz1, the fission yeast orthologue of TRF1, also has this capability (Miller et al., 2006). Mammalian TRF1 specifically mitigates lagging strand replication stress by promoting the recruitment of BLM (Bloom syndrome) (Martinez et al., 2009; Sfeir et al., 2009; Zimmermann et al., 2014), a RecQ family helicase which is capable of unwinding G4s which would impede replisome progression (Drosopoulos et al., 2015; Sun et al., 1998). A similar RecQ helicase, WRN (Werner syndrome), is also important for telomere replication as its depletion results in large telomeric deletions (Crabbe et al., 2004). WRN is capable of unwinding G4s (Drosopoulos et al., 2015; Mohaghegh et al., 2001), co-localizes at stalled replication forks with ATR and PCNA (proliferating cell nuclear antigen), a DNA clamp component of the replisome (Constantinou et al., 2000; Rodríguez-López et al., 2003), and is capable of maintaining telomeric overhangs *in vitro* in a DNA-PK-dependent manner (Kusumoto-Matsuo et al., 2010). Furthermore, both BLM and WRN have been shown to interact with TRF1, TRF2 and POT1 *in vitro*, which in most cases stimulates their helicase activity (Lillard-Wetherell et al., 2004; Opresko et al., 2005; Opresko et al. 2004; Opresko et al. 2002). The specific ability of TRF1 to recruit helicases to telomeres during S phase is modulated by post-translational modification of TRF1, including phosphorylation and poly-ADP-ribosylation (Li et al., 2018; Maresca et al., 2023).

RTEL1 (regulator of telomere elongation helicase 1) is another important regulator of telomeric replication, as it is capable of unwinding G4s and t-loops to promote replisome progression (Vannier et al., 2012; Vannier et al., 2013). RTEL1 is recruited to telomeres in S phase by TRF2 and interacts with PCNA (Sarek et al., 2015; Vannier et al., 2013). Loss of RTEL1, or inhibition of its interaction with PCNA or TRF2, results in replication fork stalling, fragile telomeres and t-loop cleavage (Sarek et al., 2015; Vannier et al., 2012; Vannier et al., 2013). These observations suggest that RTEL1 is capable of unwinding t-loops to facilitate complete replication of telomeres, which is further supported by the ability of RTEL1 to unwind t-loop-like structures *in vitro* (Youds et al., 2010).

Another way in which TRF1 and TRF2 help telomeres avoid replication stress is by the recruitment of topoisomerase IIα and the nuclease Apollo to relieve topological stress at telomeres (d'Alcontres et al., 2014; Ye et al., 2010). Furthermore, TRF1-mediated recruitment of the transcription factor and nucleotide excision repair complex TFIIH is required to suppress the fragile telomere phenotype (Yang et al., 2022). If replication fork stalling does occur, TRF1 also helps mitigate the resulting ATR-mediated DDR by virtue of its tethering TIN2/POT1/TPP1 to the telomere, which may be able to displace RPA from exposed single-stranded DNA at replication forks (Zimmermann et al., 2014). The shelterin complex thus employs a full armory of defenses against the many ways in which telomeres could provoke unwanted DDRs, particularly during DNA replication.

## 4 Replication stress is a trigger for telomere maintenance by telomerase

Shelterin proteins also ensure complete telomere maintenance through their role in recruiting the telomere lengthening enzyme telomerase. Telomerase is an evolutionarily conserved ribonucleoprotein complex (Greider and Blackburn, 1985; Morin, 1989), containing two major components: the catalytic protein subunit telomerase reverse transcriptase (hTERT in humans; Nakamura et al., 1997), and the telomerase RNA subunit (hTR in humans; Feng et al., 1995), which provides the template for *de novo* telomere synthesis. Most normal human somatic cells lack telomerase expression (Nakamura et al., 1997), and hence experience telomere shortening over successive population doublings (Harley et al., 1990), due to the inability of the conventional DNA replication machinery to copy the extreme ends of linear DNA molecules (Lingner et al., 1995; Takai et al., 2024). Approximately 85% of cancer cells overcome telomere shortening via activation of telomerase; however, telomerase is also active within highly proliferative cells, including stem and germline cells [reviewed in Bryan and Cohen (2023); Roake and Artandi (2020)]. Following synthesis of a telomeric repeat, telomerase translocates along the newly synthesized repeat to continue extending the 3′overhang of the same telomere (Greider, 1991; Wu et al., 2017). Following this, the complementary C-rich telomeric strand is filled in by the CST-Polα/primase complex [reviewed in Cai and de Lange (2023)].

Telomerase activity relies upon its successful recruitment to telomeres; this process is very tightly regulated and typically only occurs during S phase (Diede and Gottschling, 1999; Jady et al., 2006; Marcand et al., 2000; Tomlinson et al., 2006). The timing of recruitment appears to be regulated over the cell cycle, at least partially, by human TRF1 or its fission yeast orthologue Taz1, as their depletion results in accumulation of telomerase at telomeres outside S phase (Dehe et al., 2012; Tong et al., 2015). Stable recruitment of human telomerase is dependent on its interaction with TPP1, specifically an N-terminal patch termed the TEL patch within the TPP1 OB fold (Grill et al., 2018; Nandakumar et al., 2012; Zhong et al., 2012). The interaction between POT1 and TPP1 is also essential for telomerase processivity (Latrick and Cech, 2010; Wang et al., 2007), while the depletion of TPP1 and TIN2 (which bridges TPP1 and TRF1/2) results in reduced telomerase presence at telomeres (Abreu et al., 2010). The interaction of hTERT with TPP1 relies upon the TEN domain within hTERT (Schmidt et al., 2014; Stern et al., 2012), and a region within the reverse transcriptase domain (Padmanaban et al., 2023).

It has become clear that in addition to their role in ensuring accurate telomere replication, DDR proteins are also important regulators of telomerase recruitment to the telomere. In particular, the telomeric replication stress response can be considered an evolutionarily conserved trigger for bringing telomerase to those telomeres experiencing stress. The requirement for an ATR-mediated DDR for telomerase recruitment to telomeres was initially demonstrated in the fission yeast *Schizosaccharomyces pombe*, as depletion of Rad3 (the fission yeast homolog of ATR) negatively impacts telomerase recruitment in this organism (Moser et al., 2011; Moser et al., 2009; Yamazaki et al., 2012). In humans, patients with ATR mutations have poor telomere maintenance, resulting in a range of telomere abnormalities (Pennarun et al., 2010); depletion or chemical inhibition of ATR also results in reduced telomerase recruitment to telomeres in human cells (Tong et al., 2015). This may involve the 9-1-1 (Rad9-Rad1-Hus1) checkpoint complex, which is needed for full catalytic activation of ATR at single-stranded DNA (Majka et al., 2006), and also shows an association with active human telomerase (Francia et al., 2006). Inversely, induction of replication stress can promote the recruitment of telomerase and therefore its ability to lengthen telomeres. Chemical induction of replication stress using the DNA polymerase inhibitor aphidicolin results in telomere lengthening (Sfeir et al., 2009) and an ATR-dependent increased accumulation of telomerase at telomeres (Tong et al., 2015). This regulation of telomerase recruitment to telomeres by ATR appears to be highly specific to natural chromosome ends, as contrastingly it has recently been demonstrated that ATR inhibits *de novo* telomere formation by telomerase at resected DSBs (Kinzig et al., 2024). Induction of interstitial DSBs, using either Cas9 or I-SceI, resulted in telomerase-mediated *de novo* telomere formation which was significantly upregulated after inhibition of ATR, but not ATM (Kinzig et al., 2024).

As described briefly above, components of shelterin have long been known to play important roles in regulating telomere length by modulation of the access of telomerase to telomeres [reviewed in Smogorzewska and de Lange (2004)]. There have been various models proposed to provide conceptual frameworks for how this achieved; for example, the “protein counting model” postulates that the overall abundance of shelterin proteins at telomeres provides a negative regulatory signal for telomerase recruitment and/or extension (Marcand et al., 1997; Smogorzewska and de Lange, 2004). An alternative model proposes that telomerase travels along the telomere with the replication fork, and the chances of it reaching the end of the telomere are impacted by its encounters with shelterin proteins and the distance from subtelomeric origins of replication (Greider, 2016). Here, we would like to instead argue that the role of shelterin proteins in controlling telomere replication stress, discussed above, is a major component of their ability to also regulate telomerase recruitment to telomeres (Figure 1B).

As discussed above, a major function of TRF1 at telomeres is to recruit and coordinate other proteins to reduce telomeric replication stress, and TRF1 is also a major negative regulator of telomerase-mediated telomere lengthening (Smogorzewska et al., 2000). TRF1 levels at human telomeres decrease during S phase (Maresca et al., 2023; Verdun et al., 2005); this decrease is partially regulated by ATM, since experimental depletion of ATM results in accumulation of TRF1 at telomeres (Wu et al., 2007). Phosphorylation of S367 on TRF1 by ATM results in telomere elongation, due to removal of TRF1 from the telomere (McKerlie et al., 2012). This phosphorylation event also promotes telomerase recruitment to the telomere, as a phospho-null mutation of TRF1 S367 diminishes telomerase recruitment (Tong et al., 2015). Concordantly, experimental depletion or inhibition of ATM results in telomere shortening (Wu et al., 2007), and reduced recruitment of telomerase (Lee et al., 2015; Tong et al., 2015), and cells from patients with mutations in ATM have short telomeres (Metcalfe et al., 1996). Together, these data support a model in which partial depletion of TRF1 during S phase triggers telomere replication stress that results in increased telomerase recruitment. This model is supported by data from *S. pombe*, showing that deletion of the TRF1 orthologue Taz1 results in deregulated arrival of replicative polymerases at telomeres, resulting in an extended period in which both RPA and TERT are simultaneously and aberrantly recruited to telomeres, implicating a replication stress-induced ATR response in telomerase recruitment (Chang et al., 2013).

This may also be an explanation for the telomere lengthening induced by mutant versions of POT1. The first hints that POT1 is also involved in facilitating telomere replication came from the identification of familial or somatic mutations in patients with cancer; these mutations induced fragile telomeres (Calvete et al., 2015; Ramsay et al., 2013; Shi et al., 2014), and were directly shown to result in increased fork stalling in a DNA combing replication assay (Pinzaru et al., 2016). Expression of POT1 lacking its OB fold (POT1-ΔOB), rendering it incapable of binding the telomeric overhang, also results in telomeric replication stress (Pinzaru et al., 2020), which ultimately leads to increased telomerase recruitment (Laprade et al., 2020) and telomere lengthening (Loayza and De Lange, 2003; Tong et al., 2015). Consistently, the cancers that carry mutations in the POT1 OB fold also have long telomeres (Calvete et al., 2015; Ramsay et al., 2013; Robles-Espinoza et al., 2014; Shi et al., 2014). This has also been observed in mice, which possess two POT1 homologs with separation of function; the major role of POT1a is to suppress the DDR at telomeres, while POT1b also regulates telomeric overhang length (He et al., 2006; Hockemeyer et al., 2006; Wu et al., 2006). When POT1b is depleted from mouse cells, their telomeres initially shorten, triggering a DDR and an increase in ATR-mediated telomerase recruitment to telomeres (Gu et al., 2021; Takasugi et al., 2023). These cells ultimately develop ultralong telomeres as a result of the increased telomerase presence at telomeres (Takasugi et al., 2023). While physical sequestration of the 3′ telomeric overhang may contribute to the negative regulation of telomerase by POT1, it is likely that its role in preventing replication stress also contributes to the same outcome.

Another protein complex that both helps to overcome replication stress at telomeres and participates in telomere length control is the CST complex (consisting of proteins CTC1, STN1 and TEN1 in humans) (Olson and Wuttke, 2024). Human CST promotes replication restart after fork stalling; it does this through promotion of latent origin firing (Stewart et al., 2012), recruitment of the replication stress-response protein RAD51 (Chastain et al., 2016) and activation of the ATR pathway [Ackerson et al. (2020); reviewed in Olson and Wuttke (2024)]. In addition, human CST depletion causes a telomerase-dependent increase in telomere length (Chen et al., 2012). A mutation in CTC1 that leads to telomere elongation also causes an increase in the recruitment of telomerase to telomeres (Gu et al., 2018). While this may be at least partially explained by the inhibitory effect of wild-type CST on telomerase binding to its DNA substrate (Chen et al., 2012; Zaug et al., 2021), resulting in dissociation of telomerase from the telomere, it is also possible that some CTC1 mutations increase telomerase recruitment by increasing telomeric replication stress. Given that mutations in CST components cause the telomere biology disorder Coats Plus, and that these patients do not always show the telomere shortening typical of telomere biology disorders (Revy et al., 2023), further understanding of the interplay between CST, replication stress and telomerase recruitment may shed light on the telomere dysfunction underlying their disease.

## 5 Mechanisms and outcomes of replication stress-induced telomerase recruitment

Although the ways in which ATR and the replication stress response result in telomerase recruitment are incompletely understood, recent evidence implicates nuclear actin as one downstream element of this pathway. A large body of work in recent years has identified that nuclear filamentous actin (F-actin) is a key regulator of the DDR. Actin rapidly polymerizes within the nucleus in response to replication stress and DSBs; under replication stress conditions this polymerization is dependent on ATR, whose activity is required for downstream phosphorylation of another PIKK family kinase, mTOR (Lamm et al., 2020), in turn regulating F-actin through the Wiskott-Aldrich syndrome protein (WASP) family (Mok et al., 2015). F-actin facilitates the DDR by re-localization of damaged DNA to the nuclear periphery for fork restart or DNA repair (Belin et al., 2015; Caridi et al., 2018; Lamm et al., 2020; Palumbieri et al., 2023; Ryu et al., 2015). Stressed telomeres are processed by the same pathway; they have also been shown to move toward the periphery under conditions of replication stress in an F-actin-dependent manner (Lamm et al., 2020; Pinzaru et al., 2020). It has also been previously demonstrated that telomeres located closer to the nuclear periphery are more likely to be later replicating (Arnoult et al., 2010); however, it is unclear whether this timing is solely due to their position within the nucleus, or if these are stressed telomeres.

Telomerase recruitment to telomeres also requires the function of nuclear F-actin; inhibition of actin polymerization, or regulators of its polymerization or function, results in decreased telomerase recruitment under endogenous conditions in human cell lines, as well as abrogating replication stress-mediated recruitment (Harman et al., 2024). Furthermore, nuclear F-actin serves as a direct site for telomerase recruitment to stressed telomeres, which reside along these actin fibers (Harman et al., 2024). The requirement for nuclear F-actin in this process, as well as its role in telomere replication, provides a further link which connects telomerase activity to telomere replication and the DDR.

Another mechanism by which the replication stress response may increase telomerase recruitment involves the known role of ATR in promoting firing of “dormant replication origins” (Ge and Blow, 2010) within a region under replicative stress, which allows replication in problematic regions to be efficiently completed. Replication of telomeres in human cells occurs across S phase and largely originates from origins within subtelomeric regions, proceeding unidirectionally through the telomere (Drosopoulos et al., 2015; Drosopoulos et al., 2012; Higa et al., 2017a). While this appears to account for most telomeric replication, replication origins have been observed within telomeric regions (Drosopoulos et al., 2012). These origins appear to be dormant, as they are fired following replication stress in a TRF2-dependent manner (Drosopoulos et al., 2020). This likely occurs via the recruitment of ORC proteins by TRF2 (Deng et al., 2007; Higa et al., 2017b; Tatsumi et al., 2008).

There is evidence that recruitment of telomerase to telomeres needs the passage of the replication fork. In budding yeast, telomerase cannot extend an artificial minichromosome unless it contains an origin of replication (Marcand et al., 2000), and generation of a 3′ telomeric overhang (the substrate of telomerase) does not happen without passage of a replication fork (Dionne and Wellinger, 1998). In human cells, telomerase acts after the conventional replication machinery has finished replicating the rest of the telomere (Hirai et al., 2012; Zhao et al., 2009). Therefore, it is possible that increased firing of dormant replication origins within telomeres during replication stress, mediated by ATR, increases the likelihood of the replication machinery reaching the end of the telomere, triggering telomerase recruitment.

The conservation of the link between replication stress and telomerase recruitment to telomeres implies that telomerase serves a vital cellular role after such stress. Indeed, there is an increasing amount of evidence that yeast need telomerase to survive replication stress, even when global telomere length has not decreased (Chang et al., 2009; Jay et al., 2016; Noel and Wellinger, 2011; Xie et al., 2015). Replication stress can cause sudden loss of telomeres (Crabbe et al., 2004; Zhang et al., 2017), so it is likely that telomerase is needed to counteract this potentially lethal event. If the requirement for telomerase to survive replication stress extends to human cells, this could open up ways to rapidly target cancer cells by combining replication stress-inducing chemotherapeutic agents with telomerase inhibition.

## 6 Conclusion

Telomeres impose a difficult balancing act that cells must perform to maintain their proliferative capabilities and genome integrity while also avoiding aberrant telomere overextension. Cells possess a tightly controlled regulatory system which impedes the DDR from inappropriately recognizing telomeres and causing telomere loss. Concurrently, the DDR is utilized to ensure that telomeres are correctly replicated and that their length is not shortened as a byproduct of replication stress, whether exogenous or endogenous. The interactions between telomere binding proteins and the telomeric replication stress response are emerging as major factors in the maintenance of telomere lengths within defined limits, which can be perturbed in diseases such as cancer and telomere biology disorders. Further elucidation of this interplay is therefore likely to increase understanding of the mechanisms underlying these diseases.

## Data Availability

The original contributions presented in the study are included in the article; further inquiries can be directed to the corresponding author.

## References

[B1] Abreu E. Aritonovska E. Reichenbach P. Cristofari G. Culp B. Terns R. M. (2010). TIN2-tethered TPP1 recruits human telomerase to telomeres *in vivo* . Mol. Cell Biol. 30 (12), 2971–2982. 10.1128/MCB.00240-10 20404094 PMC2876666

[B2] Ackerson S. M. Gable C. I. Stewart J. A. (2020). Human CTC1 promotes TopBP1 stability and CHK1 phosphorylation in response to telomere dysfunction and global replication stress. Cell Cycle 19 (24), 3491–3507. 10.1080/15384101.2020.1849979 33269665 PMC7781613

[B3] Amiard S. Doudeau M. Pinte S. Poulet A. Lenain C. Faivre-Moskalenko C. (2007). A topological mechanism for TRF2-enhanced strand invasion. Nat. Struct. Mol. Biol. 14 (2), 147–154. 10.1038/nsmb1192 17220898

[B4] Arnoult N. Schluth-Bolard C. Letessier A. Drascovic I. Bouarich-Bourimi R. Campisi J. (2010). Replication timing of human telomeres is chromosome arm–specific, influenced by subtelomeric structures and connected to nuclear localization. PLoS Genet. 6 (4), e1000920. 10.1371/journal.pgen.1000920 20421929 PMC2858680

[B5] Baumann P. Cech T. R. (2001). Pot1, the putative telomere end-binding protein in fission yeast and humans. Science 292 (5519), 1171–1175. 10.1126/science.1060036 11349150

[B6] Belin B. J. Lee T. Mullins R. D. (2015). DNA damage induces nuclear actin filament assembly by Formin -2 and Spire-½ that promotes efficient DNA repair. Elife 4, e07735. 10.7554/eLife.07735 26287480 PMC4577826

[B7] Bilaud T. Brun C. Ancelin K. Koering C. E. Laroche T. Gilson E. (1997). Telomeric localization of TRF2, a novel human telobox protein. Nat. Genet. 17 (2), 236–239. 10.1038/ng1097-236 9326951

[B8] Blackford A. N. Jackson S. P. (2017). ATM, ATR, and DNA-PK: the trinity at the heart of the DNA damage response. Mol. Cell 66 (6), 801–817. 10.1016/j.molcel.2017.05.015 28622525

[B9] Bochman M. L. Paeschke K. Zakian V. A. (2012). DNA secondary structures: stability and function of G-quadruplex structures. Nat. Rev. Genet. 13 (11), 770–780. 10.1038/nrg3296 23032257 PMC3725559

[B10] Bonnell E. Pasquier E. Wellinger R. J. (2021). Telomere replication: solving multiple end replication problems. Front. Cell Dev. Biol. 9, 668171. 10.3389/fcell.2021.668171 33869233 PMC8047117

[B11] Broccoli D. Smogorzewska A. Chong L. de Lange T. (1997). Human telomeres contain two distinct Myb-related proteins, TRF1 and TRF2. Nat. Genet. 17 (2), 231–235. 10.1038/ng1097-231 9326950

[B12] Bryan T. M. (2020). G-quadruplexes at telomeres: friend or foe? Molecules 25 (16), 3686. 10.3390/molecules25163686 32823549 PMC7464828

[B13] Bryan T. M. Cohen S. B. (2023). “Telomerase,” in Handbook of chemical biology of nucleic acids (Springer), 1–26. 10.1007/978-981-19-9776-1_47

[B14] Cai S. W. de Lange T. (2023). CST–Polα/Primase: the second telomere maintenance machine. Genes and Dev. 37 (13-14), 555–569. 10.1101/gad.350479.123 37495394 PMC10499019

[B15] Calvete O. Martinez P. Garcia-Pavia P. Benitez-Buelga C. Paumard-Hernandez B. Fernandez V. (2015). A mutation in the *POT1* gene is responsible for cardiac angiosarcoma in *TP53*-negative Li-Fraumeni-like families. Nat. Commun. 6, 8383. 10.1038/ncomms9383 26403419 PMC4598567

[B16] Caridi C. P. D’Agostino C. Ryu T. Zapotoczny G. Delabaere L. Li X. (2018). Nuclear F-actin and myosins drive relocalization of heterochromatic breaks. Nature 559 (7712), 54–60. 10.1038/s41586-018-0242-8 29925946 PMC6051730

[B17] Celli G. B. de Lange T. (2005). DNA processing is not required for ATM-mediated telomere damage response after TRF2 deletion. Nat. Cell Biol. 7 (7), 712–718. 10.1038/ncb1275 15968270

[B18] Cesare A. J. Karlseder J. (2012). A three-state model of telomere control over human proliferative boundaries. Curr. Opin. Cell Biol. 24 (6), 731–738. 10.1016/j.ceb.2012.08.007 22947495 PMC3532573

[B19] Cesare A. J. Kaul Z. Cohen S. B. Napier C. E. Pickett H. A. Neumann A. A. (2009). Spontaneous occurrence of telomeric DNA damage response in the absence of chromosome fusions. Nat. Struct. Mol. Biol. 16 (12), 1244–1251. 10.1038/nsmb.1725 19935685

[B20] Chang M. Luke B. Kraft C. Li Z. Peter M. Lingner J. (2009). Telomerase is essential to alleviate pif1-induced replication stress at telomeres. Genetics 183 (3), 779–791. 10.1534/genetics.109.107631 19704012 PMC2778976

[B21] Chang Y.-T. Moser B. A. Nakamura T. M. (2013). Fission yeast shelterin regulates DNA polymerases and Rad3 ATR kinase to limit telomere extension. PLoS Genet. 9 (11), e1003936. 10.1371/journal.pgen.1003936 24244195 PMC3820796

[B22] Chastain M. Zhou Q. Shiva O. Whitmore L. Jia P. Dai X. (2016). Human CST facilitates genome-wide RAD51 recruitment to GC-rich repetitive sequences in response to replication stress. Cell Rep. 16 (5), 2048–2114. 10.1016/j.celrep.2016.08.008 27533181 PMC5669620

[B23] Chen L. Y. Redon S. Lingner J. (2012). The human CST complex is a terminator of telomerase activity. Nature 488, 540–544. 10.1038/nature11269 22763445

[B24] Chong L. van Steensel B. Broccoli D. Erdjument-Bromage H. Hanish J. Tempst P. (1995). A human telomeric protein. Science 270 (5242), 1663–1667. 10.1126/science.270.5242.1663 7502076

[B25] Constantinou A. Tarsounas M. Karow J. K. Brosh R. M. Bohr V. A. Hickson I. D. (2000). Werner's syndrome protein (WRN) migrates Holliday junctions and co‐localizes with RPA upon replication arrest. EMBO Rep. 1 (1), 80–84. 10.1093/embo-reports/kvd004 11256630 PMC1083680

[B26] Crabbe L. Verdun R. E. Haggblom C. I. Karlseder J. (2004). Defective telomere lagging strand synthesis in cells lacking WRN helicase activity. Science 306 (5703), 1951–1953. 10.1126/science.1103619 15591207

[B27] d'Alcontres M. S. Palacios J. A. Mejias D. Blasco M. A. (2014). TopoIIα prevents telomere fragility and formation of ultra thin DNA bridges during mitosis through TRF1-dependent binding to telomeres. Cell Cycle 13 (9), 1463–1481. 10.4161/cc.28419 24626180 PMC4050144

[B28] Davis A. J. Chen B. P. Chen D. J. (2014). DNA-PK: a dynamic enzyme in a versatile DSB repair pathway. DNA repair 17, 21–29. 10.1016/j.dnarep.2014.02.020 24680878 PMC4032623

[B29] Dehe P. M. Rog O. Ferreira M. G. Greenwood J. Cooper J. P. (2012). Taz1 enforces cell-cycle regulation of telomere synthesis. Mol. Cell 46 (6), 797–808. 10.1016/j.molcel.2012.04.022 22633956

[B30] de Lange T. (2005). Shelterin: the protein complex that shapes and safeguards human telomeres. Genes Dev. 19 (18), 2100–2110. 10.1101/gad.1346005 16166375

[B31] de Lange T. (2018). Shelterin-mediated telomere protection. Annu. Rev. Genet. 52, 223–247. 10.1146/annurev-genet-032918-021921 30208292

[B32] Denchi E. L. de Lange T. (2007). Protection of telomeres through independent control of ATM and ATR by TRF2 and POT1. Nature 448 (7157), 1068–1071. 10.1038/nature06065 17687332

[B33] Deng Z. Dheekollu J. Broccoli D. Dutta A. Lieberman P. M. (2007). The origin recognition complex localizes to telomere repeats and prevents telomere-circle formation. Curr. Biol. 17, 1989–1995. 10.1016/j.cub.2007.10.054 18006317

[B34] Diede S. J. Gottschling D. E. (1999). Telomerase-mediated telomere addition *in vivo* requires DNA primase and DNA polymerases a and d. Cell 99, 723–733. 10.1016/s0092-8674(00)81670-0 10619426

[B35] Dionne I. Wellinger R. J. (1998). Processing of telomeric DNA ends requires the passage of a replication fork. Nucleic Acids Res. 26, 5365–5371. 10.1093/nar/26.23.5365 9826760 PMC148004

[B36] Doksani Y. Wu J. Y. de Lange T. Zhuang X. (2013). Super-resolution fluorescence imaging of telomeres reveals TRF2-dependent T-loop formation. Cell 155 (2), 345–356. 10.1016/j.cell.2013.09.048 24120135 PMC4062873

[B37] Drosopoulos W. C. Deng Z. Twayana S. Kosiyatrakul S. T. Vladimirova O. Lieberman P. M. (2020). TRF2 mediates replication initiation within human telomeres to prevent telomere dysfunction. Cell Rep. 33 (6), 108379. 10.1016/j.celrep.2020.108379 33176153 PMC7790361

[B38] Drosopoulos W. C. Kosiyatrakul S. T. Schildkraut C. L. (2015). BLM helicase facilitates telomere replication during leading strand synthesis of telomeres. J. Cell Biol. 210 (2), 191–208. 10.1083/jcb.201410061 26195664 PMC4508891

[B39] Drosopoulos W. C. Kosiyatrakul S. T. Yan Z. Calderano S. G. Schildkraut C. L. (2012). Human telomeres replicate using chromosome-specific, rather than universal, replication programs. J. Cell Biol. 197 (2), 253–266. 10.1083/jcb.201112083 22508510 PMC3328383

[B40] Feng J. Funk W. D. Wang S. S. Weinrich S. L. Avilion A. A. Chiu C. P. (1995). The RNA component of human telomerase. Science 269 (5228), 1236–1241. 10.1126/science.7544491 7544491

[B41] Fouche N. Ozgur S. Roy D. Griffith J. D. (2006). Replication fork regression in repetitive DNAs. Nucleic Acids Res. 34 (20), 6044–6050. 10.1093/nar/gkl757 17071963 PMC1635326

[B42] Francia S. Weiss R. S. Hande M. P. Freire R. di Fagagna F. d. A. (2006). Telomere and telomerase modulation by the mammalian Rad9/Rad1/Hus1 DNA-damage-checkpoint complex. Curr. Biol. 16 (15), 1551–1558. 10.1016/j.cub.2006.06.066 16890531

[B43] Ge X. Q. Blow J. J. (2010). Chk1 inhibits replication factory activation but allows dormant origin firing in existing factories. J. Cell Biol. 191 (7), 1285–1297. 10.1083/jcb.201007074 21173116 PMC3010067

[B44] Gong Y. de Lange T. (2010). A Shld1-controlled POT1a provides support for repression of ATR signaling at telomeres through RPA exclusion. Mol. Cell 40 (3), 377–387. 10.1016/j.molcel.2010.10.016 21070964 PMC3111920

[B45] Gottlieb T. M. Jackson S. P. (1993). The DNA-dependent protein kinase: requirement for DNA ends and association with Ku antigen. Cell 72 (1), 131–142. 10.1016/0092-8674(93)90057-w 8422676

[B46] Greider C. W. (1991). Telomerase is processive. Mol. Cell. Biol. 11 (9), 4572–4580. 10.1128/mcb.11.9.4572 1875940 PMC361337

[B47] Greider C. W. (2016). Regulating telomere length from the inside out: the replication fork model. Genes Dev. 30 (13), 1483–1491. 10.1101/gad.280578.116 27401551 PMC4949321

[B48] Greider C. W. Blackburn E. H. (1985). Identification of a specific telomere terminal transferase activity in Tetrahymena extracts. Cell 43 (2 Pt 1), 405–413. 10.1016/0092-8674(85)90170-9 3907856

[B49] Griffith J. D. Comeau L. Rosenfield S. Stansel R. M. Bianchi A. Moss H. (1999). Mammalian telomeres end in a large duplex loop. Cell 97 (4), 503–514. 10.1016/s0092-8674(00)80760-6 10338214

[B50] Grill S. Tesmer V. M. Nandakumar J. (2018). The N terminus of the OB domain of telomere protein TPP1 is critical for telomerase action. Cell Rep. 22 (5), 1132–1140. 10.1016/j.celrep.2018.01.012 29386102 PMC5815312

[B51] Gu P. Jia S. Takasugi T. Smith E. Nandakumar J. Hendrickson E. (2018). CTC1-STN1 coordinates G- and C-strand synthesis to regulate telomere length. Aging Cell 17, e12783. 10.1111/acel.12783 29774655 PMC6052479

[B52] Gu P. Jia S. Takasugi T. Tesmer V. M. Nandakumar J. Chen Y. (2021). Distinct functions of POT1 proteins contribute to the regulation of telomerase recruitment to telomeres. Nat. Commun. 12 (1), 5514. 10.1038/s41467-021-25799-7 34535663 PMC8448735

[B53] Harley C. B. Futcher A. B. Greider C. W. (1990). Telomeres shorten during ageing of human fibroblasts. Nature 345 (6274), 458–460. 10.1038/345458a0 2342578

[B54] Harman A. Kartawinata M. Maroun N. M. Nguyen D. R. Hughes W. E. Winardi K. (2024). Nuclear actin and DNA replication stress regulate the recruitment of human telomerase to telomeres. bioRxiv. 2024.03. 25.586711. 10.1101/2024.03.25.586711 PMC12672677 41331243

[B55] Harrison J. C. Haber J. E. (2006). Surviving the breakup: the DNA damage checkpoint. Annu. Rev. Genet. 40, 209–235. 10.1146/annurev.genet.40.051206.105231 16805667

[B56] He H. Multani A. S. Cosme-Blanco W. Tahara H. Ma J. Pathak S. (2006). POT1b protects telomeres from end-to-end chromosomal fusions and aberrant homologous recombination. EMBO J. 25, 5180–5190. 10.1038/sj.emboj.7601294 17053789 PMC1630418

[B57] Higa M. Fujita M. Yoshida K. (2017a). DNA replication origins and fork progression at mammalian telomeres. Genes 8 (4), 112. 10.3390/genes8040112 28350373 PMC5406859

[B58] Higa M. Kushiyama T. Kurashige S. Kohmon D. Enokitani K. Iwahori S. (2017b). TRF2 recruits ORC through TRFH domain dimerization. Biochim. Biophys. Acta Mol. Cell Res. 1864 (1), 191–201. 10.1016/j.bbamcr.2016.11.004 27836746

[B59] Hirai Y. Masutomi K. Ishikawa F. (2012). Kinetics of DNA replication and telomerase reaction at a single-seeded telomere in human cells. Genes cells. 17, 186–204. 10.1111/j.1365-2443.2012.01581.x 22353550

[B60] Hockemeyer D. Daniels J. P. Takai H. de Lange T. (2006). Recent expansion of the telomeric complex in rodents: two distinct POT1 proteins protect mouse telomeres. Cell 126 (1), 63–77. 10.1016/j.cell.2006.04.044 16839877

[B61] Hockemeyer D. Palm W. Else T. Daniels J. P. Takai K. K. Ye J. Z. (2007). Telomere protection by mammalian Pot1 requires interaction with Tpp1. Nat. Struct. Mol. Biol. 14 (8), 754–761. 10.1038/nsmb1270 17632522

[B62] Houghtaling B. R. Cuttonaro L. Chang W. Smith S. (2004). A dynamic molecular link between the telomere length regulator TRF1 and the chromosome end protector TRF2. Curr. Biol. 14 (18), 1621–1631. 10.1016/j.cub.2004.08.052 15380063

[B63] Ivessa A. S. Zhou J.-Q. Schulz V. P. Monson E. K. Zakian V. A. (2002). Saccharomyces Rrm3p, a 5′ to 3′ DNA helicase that promotes replication fork progression through telomeric and subtelomeric DNA. Genes and Dev. 16 (11), 1383–1396. 10.1101/gad.982902 12050116 PMC186315

[B64] Jady B. E. Richard P. Bertrand E. Kiss T. (2006). Cell cycle-dependent recruitment of telomerase RNA and Cajal bodies to human telomeres. Mol. Biol. Cell 17 (2), 944–954. 10.1091/mbc.e05-09-0904 16319170 PMC1356602

[B65] Jay K. A. Smith D. L. Blackburn E. H. (2016). Early loss of telomerase action in yeast creates a dependence on the DNA damage response adaptor proteins. Mol. Cell Biol. 36 (14), 1908–1919. 10.1128/MCB.00943-15 27161319 PMC4936065

[B66] Karlseder J. Hoke K. Mirzoeva O. K. Bakkenist C. Kastan M. B. Petrini J. H. (2004). The telomeric protein TRF2 binds the ATM kinase and can inhibit the ATM-dependent DNA damage response. PLoS Biol. 2 (8), E240. 10.1371/journal.pbio.0020240 15314656 PMC509302

[B67] Kim S. H. Kaminker P. Campisi J. (1999). TIN2, a new regulator of telomere length in human cells. Nat. Genet. 23 (4), 405–412. 10.1038/70508 10581025 PMC4940194

[B68] Kinzig C. G. Zakusilo G. Takai K. K. Myler L. R. de Lange T. (2024). ATR blocks telomerase from converting DNA breaks into telomeres. Science 383 (6684), 763–770. 10.1126/science.adg3224 38359122 PMC11267623

[B69] Kusumoto-Matsuo R. Opresko P. L. Ramsden D. Tahara H. Bohr V. A. (2010). Cooperation of DNA-PKcs and WRN helicase in the maintenance of telomeric D-loops. Aging (Albany NY) 2 (5), 274–284. 10.18632/aging.100141 20519774 PMC2898018

[B70] Lamm N. Read M. N. Nobis M. Van Ly D. Page S. G. Masamsetti V. P. (2020). Nuclear F-actin counteracts nuclear deformation and promotes fork repair during replication stress. Nat. Cell Biol. 22 (12), 1460–1470. 10.1038/s41556-020-00605-6 33257806

[B71] Laprade H. Querido E. Smith M. J. Guerit D. Crimmins H. Conomos D. (2020). Single-molecule imaging of telomerase RNA reveals a recruitment-retention model for telomere elongation. Mol. Cell 79 (1), 115–126. 10.1016/j.molcel.2020.05.005 32497497

[B72] Latrick C. M. Cech T. R. (2010). POT1-TPP1 enhances telomerase processivity by slowing primer dissociation and aiding translocation. EMBO J. 29 (5), 924–933. 10.1038/emboj.2009.409 20094033 PMC2837173

[B73] Lee J.-H. Paull T. T. (2005). ATM activation by DNA double-strand breaks through the Mre11-Rad50-Nbs1 complex. Science 308 (5721), 551–554. 10.1126/science.1108297 15790808

[B74] Lee S. S. Bohrson C. Pike A. M. Wheelan S. J. Greider C. W. (2015). ATM kinase is required for telomere elongation in mouse and human cells. Cell Rep. 13 (8), 1623–1632. 10.1016/j.celrep.2015.10.035 26586427 PMC4663052

[B75] Li B. Oestreich S. de Lange T. (2000). Identification of human Rap1: implications for telomere evolution. Cell 101 (5), 471–483. 10.1016/s0092-8674(00)80858-2 10850490

[B76] Li F. Kim H. Ji Z. Zhang T. Chen B. Ge Y. (2018). The BUB3-BUB1 complex promotes telomere DNA replication. Mol. Cell 70 (3), 395–407. 10.1016/j.molcel.2018.03.032 29727616 PMC5982595

[B77] Lillard-Wetherell K. Machwe A. Langland G. T. Combs K. A. Behbehani G. K. Schonberg S. A. (2004). Association and regulation of the BLM helicase by the telomere proteins TRF1 and TRF2. Hum. Mol. Genet. 13 (17), 1919–1932. 10.1093/hmg/ddh193 15229185

[B78] Lingner J. Cooper J. P. Cech T. R. (1995). Telomerase and DNA end replication: no longer a lagging strand problem? Science 269 (5230), 1533–1534. 10.1126/science.7545310 7545310

[B79] Liu D. Safari A. O'Connor M. S. Chan D. W. Laegeler A. Qin J. (2004). PTOP interacts with POT1 and regulates its localization to telomeres. Nat. Cell Biol. 6 (7), 673–680. 10.1038/ncb1142 15181449

[B80] Loayza D. De Lange T. (2003). POT1 as a terminal transducer of TRF1 telomere length control. Nature 423 (6943), 1013–1018. 10.1038/nature01688 12768206

[B81] Majka J. Niedziela-Majka A. Burgers P. M. (2006). The checkpoint clamp activates Mec1 kinase during initiation of the DNA damage checkpoint. Mol. Cell 24 (6), 891–901. 10.1016/j.molcel.2006.11.027 17189191 PMC1850967

[B82] Makarov V. L. Hirose Y. Langmore J. P. (1997). Long G tails at both ends of human chromosomes suggest a C strand degradation mechanism for telomere shortening. Cell 88 (5), 657–666. 10.1016/s0092-8674(00)81908-x 9054505

[B83] Makovets S. Herskowitz I. Blackburn E. H. (2004). Anatomy and dynamics of DNA replication fork movement in yeast telomeric regions. Mol. Cell Biol. 24 (9), 4019–4031. 10.1128/MCB.24.9.4019-4031.2004 15082794 PMC387773

[B84] Marcand S. Brevet V. Mann C. Gilson E. (2000). Cell cycle restriction of telomere elongation. Curr. Biol. 10, 487–490. 10.1016/s0960-9822(00)00450-4 10801419

[B85] Marcand S. Gilson E. Shore D. (1997). A protein-counting mechanism for telomere length regulation in yeast. Science 275 (5302), 986–990. 10.1126/science.275.5302.986 9020083

[B86] Maresca C. Dello Stritto A. D’Angelo C. Petti E. Rizzo A. Vertecchi E. (2023). PARP1 allows proper telomere replication through TRF1 poly (ADP-ribosyl) ation and helicase recruitment. Commun. Biol. 6 (1), 234. 10.1038/s42003-023-04596-6 36864251 PMC9981704

[B87] Martinez P. Thanasoula M. Munoz P. Liao C. Tejera A. McNees C. (2009). Increased telomere fragility and fusions resulting from TRF1 deficiency lead to degenerative pathologies and increased cancer in mice. Genes Dev. 23 (17), 2060–2075. 10.1101/gad.543509 19679647 PMC2751970

[B88] McClintock B. (1941). The stability of broken ends of chromosomes in zea mays. Genetics 26 (2), 234–282. 10.1093/genetics/26.2.234 17247004 PMC1209127

[B89] McElligott R. Wellinger R. J. (1997). The terminal DNA structure of mammalian chromosomes. EMBO J. 16 (12), 3705–3714. 10.1093/emboj/16.12.3705 9218811 PMC1169994

[B90] McKerlie M. Lin S. Zhu X.-D. (2012). ATM regulates proteasome-dependent subnuclear localization of TRF1, which is important for telomere maintenance. Nucleic acids Res. 40 (9), 3975–3989. 10.1093/nar/gks035 22266654 PMC3351164

[B91] McNees C. J. Tejera A. M. Martínez P. Murga M. Mulero F. Fernandez-Capetillo O. (2010). ATR suppresses telomere fragility and recombination but is dispensable for elongation of short telomeres by telomerase. J. Cell Biol. 188 (5), 639–652. 10.1083/jcb.200908136 20212315 PMC2835929

[B92] Metcalfe J. A. Parkhill J. Campbell L. Stacey M. Biggs P. Byrd P. J. (1996). Accelerated telomere shortening in ataxia telangiectasia. Nat. Genet. 13 (3), 350–353. 10.1038/ng0796-350 8673136

[B93] Miller K. M. Rog O. Cooper J. P. (2006). Semi-conservative DNA replication through telomeres requires Taz1. Nature 440 (7085), 824–828. 10.1038/nature04638 16598261

[B94] Mohaghegh P. Karow J. K. Brosh Jr R. M. Bohr V. A. Hickson I. D. (2001). The Bloom’s and Werner’s syndrome proteins are DNA structure-specific helicases. Nucleic acids Res. 29 (13), 2843–2849. 10.1093/nar/29.13.2843 11433031 PMC55766

[B95] Mok K.-W. Chen H. Lee W. M. Cheng C. Y. (2015). rpS6 regulates blood-testis barrier dynamics through Arp3-mediated actin microfilament organization in rat Sertoli cells. An *in vitro* study. Endocrinology 156 (5), 1900–1913. 10.1210/en.2014-1791 25714812 PMC4398761

[B96] Morin G. B. (1989). The human telomere terminal transferase enzyme is a ribonucleoprotein that synthesizes TTAGGG repeats. Cell 59 (3), 521–529. 10.1016/0092-8674(89)90035-4 2805070

[B97] Moser B. A. Chang Y. T. Kosti J. Nakamura T. M. (2011). Tel1ATM and Rad3ATR kinases promote Ccq1-Est1 interaction to maintain telomeres in fission yeast. Nat. Struct. Mol. Biol. 18 (12), 1408–1413. 10.1038/nsmb.2187 22101932 PMC3230746

[B98] Moser B. A. Subramanian L. Khair L. Chang Y. T. Nakamura T. M. (2009). Fission yeast Tel1(ATM) and Rad3(ATR) promote telomere protection and telomerase recruitment. PLoS Genet. 5 (8), e1000622. 10.1371/journal.pgen.1000622 19714219 PMC2726628

[B99] Moyzis R. K. Buckingham J. M. Cram L. S. Dani M. Deaven L. L. Jones M. D. (1988). A highly conserved repetitive DNA sequence, (TTAGGG)_n_, present at the telomeres of human chromosomes. Proc. Natl. Acad. Sci. U. S. A. 85, 6622–6626. 10.1073/pnas.85.18.6622 3413114 PMC282029

[B100] Muller H. J. (1938). The remaking of chromosomes. Collect. Net. 8, 198.

[B101] Nakamura T. M. Morin G. B. Chapman K. B. Weinrich S. L. Andrews W. H. Lingner J. (1997). Telomerase catalytic subunit homologs from fission yeast and human. Science 277 (5328), 955–959. 10.1126/science.277.5328.955 9252327

[B102] Nandakumar J. Bell C. F. Weidenfeld I. Zaug A. J. Leinwand L. A. Cech T. R. (2012). The TEL patch of telomere protein TPP1 mediates telomerase recruitment and processivity. Nature 492 (7428), 285–289. 10.1038/nature11648 23103865 PMC3521872

[B103] Noel J. F. Wellinger R. J. (2011). Abrupt telomere losses and reduced end-resection can explain accelerated senescence of Smc5/6 mutants lacking telomerase. DNA Repair (Amst. ) 10, 271–282. 10.1016/j.dnarep.2010.11.010 21190904

[B104] Nowsheen S. Yang E. (2012). The intersection between DNA damage response and cell death pathways. Exp. Oncol. 34 (3), 243–254. 23070009 PMC3754840

[B105] Ohki R. Ishikawa F. (2004). Telomere-bound TRF1 and TRF2 stall the replication fork at telomeric repeats. Nucleic Acids Res. 32 (5), 1627–1637. 10.1093/nar/gkh309 15007108 PMC390322

[B106] Okamoto K. Bartocci C. Ouzounov I. Diedrich J. K. Yates J. R. Denchi E. L. (2013). A two-step mechanism for TRF2-mediated chromosome-end protection. Nature 494 (7438), 502–505. 10.1038/nature11873 23389450 PMC3733551

[B107] Olson C. L. Wuttke D. S. (2024). Guardians of the genome: how the single-stranded DNA-binding proteins RPA and CST facilitate telomere replication. Biomolecules 14 (3), 263. 10.3390/biom14030263 38540683 PMC10968030

[B108] Opresko P. L. Mason P. A. Podell E. R. Lei M. Hickson I. D. Cech T. R. (2005). POT1 stimulates RecQ helicases WRN and BLM to unwind telomeric DNA substrates. J. Biol. Chem. 280 (37), 32069–32080. 10.1074/jbc.M505211200 16030011

[B109] Opresko P. L. Otterlei M. Graakjaer J. Bruheim P. Dawut L. Kolvraa S. (2004). The Werner syndrome helicase and exonuclease cooperate to resolve telomeric D loops in a manner regulated by TRF1 and TRF2. Mol. Cell 14 (6), 763–774. 10.1016/j.molcel.2004.05.023 15200954

[B110] Opresko P. L. von Kobbe C. Laine J. P. Harrigan J. Hickson I. D. Bohr V. A. (2002). Telomere-binding protein TRF2 binds to and stimulates the Werner and Bloom syndrome helicases. J. Biol. Chem. 277 (43), 41110–41119. 10.1074/jbc.M205396200 12181313

[B111] Padmanaban S. Tesmer V. M. Nandakumar J. (2023). Interaction hub critical for telomerase recruitment and primer-template handling for catalysis. Life Sci. Alliance 6 (6), e202201727. 10.26508/lsa.202201727 36963832 PMC10055720

[B112] Palumbieri M. D. Merigliano C. González-Acosta D. Kuster D. Krietsch J. Stoy H. (2023). Nuclear actin polymerization rapidly mediates replication fork remodeling upon stress by limiting PrimPol activity. Nat. Commun. 14 (1), 7819. 10.1038/s41467-023-43183-5 38016948 PMC10684888

[B113] Pennarun G. Hoffschir F. Revaud D. Granotier C. Gauthier L. R. Mailliet P. (2010). ATR contributes to telomere maintenance in human cells. Nucleic acids Res. 38 (9), 2955–2963. 10.1093/nar/gkp1248 20147462 PMC2874998

[B114] Pinzaru A. M. Hom R. A. Beal A. Phillips A. F. Ni E. Cardozo T. (2016). Telomere replication stress induced by POT1 inactivation accelerates tumorigenesis. Cell Rep. 15 (10), 2170–2184. 10.1016/j.celrep.2016.05.008 27239034 PMC6145145

[B115] Pinzaru A. M. Kareh M. Lamm N. Lazzerini-Denchi E. Cesare A. J. Sfeir A. (2020). Replication stress conferred by POT1 dysfunction promotes telomere relocalization to the nuclear pore. Genes Dev. 34 (23-24), 1619–1636. 10.1101/gad.337287.120 33122293 PMC7706707

[B116] Ramsay A. J. Quesada V. Foronda M. Conde L. Martinez-Trillos A. Villamor N. (2013). POT1 mutations cause telomere dysfunction in chronic lymphocytic leukemia. Nat. Genet. 45 (5), 526–530. 10.1038/ng.2584 23502782

[B117] Revy P. Kannengiesser C. Bertuch A. A. (2023). Genetics of human telomere biology disorders. Nat. Rev. Genet. 24 (2), 86–108. 10.1038/s41576-022-00527-z 36151328

[B118] Roake C. M. Artandi S. E. (2020). Regulation of human telomerase in homeostasis and disease. Nat. Rev. Mol. Cell Biol. 21 (7), 384–397. 10.1038/s41580-020-0234-z 32242127 PMC7377944

[B119] Robles-Espinoza C. D. Harland M. Ramsay A. J. Aoude L. G. Quesada V. Ding Z. (2014). *POT1* loss-of-function variants predispose to familial melanoma. Nat. Genet. 46, 478–481. 10.1038/ng.2947 24686849 PMC4266105

[B120] Rodríguez-López A. M. Jackson D. A. Nehlin J. O. Iborra F. Warren A. V. Cox L. S. (2003). Characterisation of the interaction between WRN, the helicase/exonuclease defective in progeroid Werner's syndrome, and an essential replication factor, PCNA. Mech. ageing Dev. 124 (2), 167–174. 10.1016/s0047-6374(02)00131-8 12633936

[B121] Ryu T. Spatola B. Delabaere L. Bowlin K. Hopp H. Kunitake R. (2015). Heterochromatic breaks move to the nuclear periphery to continue recombinational repair. Nat. Cell Biol. 17 (11), 1401–1411. 10.1038/ncb3258 26502056 PMC4628585

[B122] Saldivar J. C. Cortez D. Cimprich K. A. (2017). The essential kinase ATR: ensuring faithful duplication of a challenging genome. Nat. Rev. Mol. Cell Biol. 18 (10), 622–636. 10.1038/nrm.2017.67 28811666 PMC5796526

[B123] Sarek G. Vannier J. B. Panier S. Petrini J. H. J. Boulton S. J. (2015). TRF2 recruits RTEL1 to telomeres in S phase to promote t-loop unwinding. Mol. Cell 57 (4), 622–635. 10.1016/j.molcel.2014.12.024 25620558 PMC4339303

[B124] Schmidt J. C. Dalby A. B. Cech T. R. (2014). Identification of human TERT elements necessary for telomerase recruitment to telomeres. Elife 3, e03563. 10.7554/eLife.03563 25271372 PMC4359370

[B125] Sfeir A. de Lange T. (2012). Removal of shelterin reveals the telomere end-protection problem. Science 336 (6081), 593–597. 10.1126/science.1218498 22556254 PMC3477646

[B126] Sfeir A. Kabir S. van Overbeek M. Celli G. B. de Lange T. (2010). Loss of Rap1 induces telomere recombination in the absence of NHEJ or a DNA damage signal. Science 327 (5973), 1657–1661. 10.1126/science.1185100 20339076 PMC2864730

[B127] Sfeir A. Kosiyatrakul S. T. Hockemeyer D. MacRae S. L. Karlseder J. Schildkraut C. L. (2009). Mammalian telomeres resemble fragile sites and require TRF1 for efficient replication. Cell 138 (1), 90–103. 10.1016/j.cell.2009.06.021 19596237 PMC2723738

[B128] Shechter D. Costanzo V. Gautier J. (2004). Regulation of DNA replication by ATR: signaling in response to DNA intermediates. DNA Repair (Amst) 3 (8-9), 901–908. 10.1016/j.dnarep.2004.03.020 15279775

[B129] Shi J. Yang X. R. Ballew B. Rotunno M. Calista D. Fargnoli M. C. (2014). Rare missense variants in *POT1* predispose to familial cutaneous malignant melanoma. Nat. Genet. 46, 482–486. 10.1038/ng.2941 24686846 PMC4056593

[B130] Shiloh Y. Ziv Y. (2013). The ATM protein kinase: regulating the cellular response to genotoxic stress, and more. Nat. Rev. Mol. Cell Biol. 14 (4), 197–210. 10.1038/nrm3546 23847781

[B131] Smogorzewska A. de Lange T. (2004). Regulation of telomerase by telomeric proteins. Annu. Rev. Biochem. 73, 177–208. 10.1146/annurev.biochem.73.071403.160049 15189140

[B132] Smogorzewska A. van Steensel B. Bianchi A. Oelmann S. Schaefer M. R. Schnapp G. (2000). Control of human telomere length by TRF1 and TRF2. Mol. Cell Biol. 20 (5), 1659–1668. 10.1128/MCB.20.5.1659-1668.2000 10669743 PMC85349

[B133] Stansel R. M. de Lange T. Griffith J. D. (2001). T-loop assembly *in vitro* involves binding of TRF2 near the 3' telomeric overhang. EMBO J. 20 (19), 5532–5540. 10.1093/emboj/20.19.5532 11574485 PMC125642

[B134] Stern J. L. Zyner K. G. Pickett H. A. Cohen S. B. Bryan T. M. (2012). Telomerase recruitment requires both TCAB1 and Cajal bodies independently. Mol. Cell Biol. 32 (13), 2384–2395. 10.1128/MCB.00379-12 22547674 PMC3434490

[B135] Stewart J. A. Wang F. Chaiken M. F. Kasbek C. Chastain P. D. Wright W. E. (2012). Human CST promotes telomere duplex replication and general replication restart after fork stalling. Embo J. 31 (17), 3537–3549. 10.1038/emboj.2012.215 22863775 PMC3433780

[B136] Sun H. Karow J. K. Hickson I. D. Maizels N. (1998). The Bloom's syndrome helicase unwinds G4 DNA. J. Biol. Chem. 273 (42), 27587–27592. 10.1074/jbc.273.42.27587 9765292

[B137] Takai H. Aria V. Borges P. Yeeles J. T. P. de Lange T. (2024). CST–polymerase α-primase solves a second telomere end-replication problem. Nature 627 (8004), 664–670. 10.1038/s41586-024-07137-1 38418884 PMC11160940

[B138] Takai K. K. Kibe T. Donigian J. R. Frescas D. de Lange T. (2011). Telomere protection by TPP1/POT1 requires tethering to TIN2. Mol. Cell 44 (4), 647–659. 10.1016/j.molcel.2011.08.043 22099311 PMC3222871

[B139] Takasugi T. Gu P. Liang F. Staco I. Chang S. (2023). *Pot1b−/−* tumors activate G-quadruplex-induced DNA damage to promote telomere hyper-elongation. Nucleic Acids Res. 51 (17), 9227–9247. 10.1093/nar/gkad648 37560909 PMC10516629

[B140] Tatsumi Y. Ezura K. Yoshida K. Yugawa T. Narisawa-Saito M. Kiyono T. (2008). Involvement of human ORC and TRF2 in pre-replication complex assembly at telomeres. Genes cells. 13 (10), 1045–1059. 10.1111/j.1365-2443.2008.01224.x 18761675

[B141] Tomlinson R. L. Ziegler T. D. Supakorndej T. Terns R. M. Terns M. P. (2006). Cell cycle-regulated trafficking of human telomerase to telomeres. Mol. Biol. Cell 17 (2), 955–965. 10.1091/mbc.e05-09-0903 16339074 PMC1356603

[B142] Tong A. S. Stern J. L. Sfeir A. Kartawinata M. de Lange T. Zhu X. D. (2015). ATM and ATR signaling regulate the recruitment of human telomerase to telomeres. Cell Rep. 13 (8), 1633–1646. 10.1016/j.celrep.2015.10.041 26586433 PMC4662887

[B143] Van Ly D. Low R. R. J. Frolich S. Bartolec T. K. Kafer G. R. Pickett H. A. (2018). Telomere loop dynamics in chromosome end protection. Mol. Cell 71 (4), 510–525. 10.1016/j.molcel.2018.06.025 30033372

[B144] Vannier J.-B. Pavicic-Kaltenbrunner V. Petalcorin M. I. Ding H. Boulton S. J. (2012). RTEL1 dismantles T loops and counteracts telomeric G4-DNA to maintain telomere integrity. Cell 149 (4), 795–806. 10.1016/j.cell.2012.03.030 22579284

[B145] Vannier J.-B. Sandhu S. Petalcorin M. I. Wu X. Nabi Z. Ding H. (2013). RTEL1 is a replisome-associated helicase that promotes telomere and genome-wide replication. Science 342 (6155), 239–242. 10.1126/science.1241779 24115439

[B146] Verdun R. E. Crabbe L. Haggblom C. Karlseder J. (2005). Functional human telomeres are recognized as DNA damage in G2 of the cell cycle. Mol. Cell 20 (4), 551–561. 10.1016/j.molcel.2005.09.024 16307919

[B147] Verdun R. E. Karlseder J. (2006). The DNA damage machinery and homologous recombination pathway act consecutively to protect human telomeres. Cell 127 (4), 709–720. 10.1016/j.cell.2006.09.034 17110331

[B148] Wang F. Podell E. R. Zaug A. J. Yang Y. Baciu P. Cech T. R. (2007). The POT1-TPP1 telomere complex is a telomerase processivity factor. Nature 445 (7127), 506–510. 10.1038/nature05454 17237768

[B149] Wu L. Multani A. S. He H. Cosme-Blanco W. Deng Y. Deng J. M. (2006). Pot1 deficiency initiates DNA damage checkpoint activation and aberrant homologous recombination at telomeres. Cell 126, 49–62. 10.1016/j.cell.2006.05.037 16839876

[B150] Wu R. A. Upton H. E. Vogan J. M. Collins K. (2017). Telomerase mechanism of telomere synthesis. Annu. Rev. Biochem. 86 (1), 439–460. 10.1146/annurev-biochem-061516-045019 28141967 PMC5812681

[B151] Wu Y. Xiao S. Zhu X. D. (2007). MRE11-RAD50-NBS1 and ATM function as co-mediators of TRF1 in telomere length control. Nat. Struct. Mol. Biol. 14 (9), 832–840. 10.1038/nsmb1286 17694070

[B152] Xie Z. Jay K. A. Smith D. L. Zhang Y. Liu Z. Zheng J. (2015). Early telomerase inactivation accelerates aging independently of telomere length. Cell 160 (5), 928–939. 10.1016/j.cell.2015.02.002 25723167 PMC4496004

[B153] Yamazaki H. Tarumoto Y. Ishikawa F. (2012). Tel1(ATM) and Rad3(ATR) phosphorylate the telomere protein Ccq1 to recruit telomerase and elongate telomeres in fission yeast. Genes Dev. 26 (3), 241–246. 10.1101/gad.177873.111 22302936 PMC3278891

[B154] Yang J. Xu Z. P. Huang Y. Hamrick H. E. Duerksen-Hughes P. J. Yu Y. N. (2004). ATM and ATR: sensing DNA damage. World J. Gastroenterol. 10 (2), 155–160. 10.3748/wjg.v10.i2.155 14716813 PMC4716994

[B155] Yang Z. Sharma K. de Lange T. (2022). TRF1 uses a noncanonical function of TFIIH to promote telomere replication. Genes Dev. 36 (17-18), 956–969. 10.1101/gad.349975.122 36229075 PMC9732906

[B156] Ye J. Lenain C. Bauwens S. Rizzo A. Saint-Leger A. Poulet A. (2010). TRF2 and apollo cooperate with topoisomerase 2alpha to protect human telomeres from replicative damage. Cell 142 (2), 230–242. 10.1016/j.cell.2010.05.032 20655466

[B157] Ye J. Z. de Lange T. (2004). TIN2 is a tankyrase 1 PARP modulator in the TRF1 telomere length control complex. Nat. Genet. 36 (6), 618–623. 10.1038/ng1360 15133513

[B158] Ye J. Z. Donigian J. R. van Overbeek M. Loayza D. Luo Y. Krutchinsky A. N. (2004a). TIN2 binds TRF1 and TRF2 simultaneously and stabilizes the TRF2 complex on telomeres. J. Biol. Chem. 279 (45), 47264–47271. 10.1074/jbc.M409047200 15316005

[B159] Ye J. Z. Hockemeyer D. Krutchinsky A. N. Loayza D. Hooper S. M. Chait B. T. (2004b). POT1-interacting protein PIP1: a telomere length regulator that recruits POT1 to the TIN2/TRF1 complex. Genes Dev. 18 (14), 1649–1654. 10.1101/gad.1215404 15231715 PMC478187

[B160] Youds J. L. Mets D. G. McIlwraith M. J. Martin J. S. Ward J. D. Nj O. N. (2010). RTEL-1 enforces meiotic crossover interference and homeostasis. Science 327 (5970), 1254–1258. 10.1126/science.1183112 20203049 PMC4770885

[B161] Zaug A. J. Lim C. J. Olson C. L. Carilli M. T. Goodrich K. J. Wuttke D. S. (2021). CST does not evict elongating telomerase but prevents initiation by ssDNA binding. Nucleic Acids Res. 49 (20), 11653–11665. 10.1093/nar/gkab942 34718732 PMC8599947

[B162] Zhang T. Zhang Z. Li F. Hu Q. Liu H. Tang M. (2017). Looping-out mechanism for resolution of replicative stress at telomeres. EMBO Rep. 18 (8), 1412–1428. 10.15252/embr.201643866 28615293 PMC5538764

[B163] Zhao Y. Hoshiyama H. Shay J. W. Wright W. E. (2008). Quantitative telomeric overhang determination using a double-strand specific nuclease. Nucleic Acids Res. 36, e14. 10.1093/nar/gkm1063 18073199 PMC2241892

[B164] Zhao Y. Sfeir A. J. Zou Y. Buseman C. M. Chow T. T. Shay J. W. (2009). Telomere extension occurs at most chromosome ends and is uncoupled from fill-in in human cancer cells. Cell 138 (3), 463–475. 10.1016/j.cell.2009.05.026 19665970 PMC2726829

[B165] Zhong F. L. Batista L. F. Freund A. Pech M. F. Venteicher A. S. Artandi S. E. (2012). TPP1 OB-fold domain controls telomere maintenance by recruiting telomerase to chromosome ends. Cell 150 (3), 481–494. 10.1016/j.cell.2012.07.012 22863003 PMC3516183

[B166] Zimmermann M. Kibe T. Kabir S. de Lange T. (2014). TRF1 negotiates TTAGGG repeat-associated replication problems by recruiting the BLM helicase and the TPP1/POT1 repressor of ATR signaling. Genes Dev. 28 (22), 2477–2491. 10.1101/gad.251611.114 25344324 PMC4233241

[B167] Zou L. Elledge S. J. (2003). Sensing DNA damage through ATRIP recognition of RPA-ssDNA complexes. Science 300 (5625), 1542–1548. 10.1126/science.1083430 12791985

